# Dr. Pardon Leland Kimball

**Published:** 1912-10

**Authors:** 


Dr. Pardon Leland Kimball, Buffalo, 1880, a Civil War veteran who had practiced in Illinois and Iowa, died this summer at his late residence in Long Beach, Calif., aged 71.

